# Corporate Social Responsibility and Energy-Related Pro-Environmental Behaviour of Employees in Hospitality Industry

**DOI:** 10.3390/ijerph192316141

**Published:** 2022-12-02

**Authors:** Huan Zhang, Khaoula Omhand, Huaizheng Li, Aqeel Ahmad, Sarminah Samad, Darie Gavrilut, Daniel Badulescu

**Affiliations:** 1 Business School, Guangdong Ocean University, Yangjiang 529599, China; 2 Business School, Staffordshire University, Leek Road, Stoke-on-Trent ST4 2DF, UK; 3 School of Marxism, University of International Business and Economics, Beijing 100029, China; 4 Faculty of Management Sciences, University of Central Punjab, Lahore 54000, Pakistan; 5 Department of Business Administration, College of Business and Administration, Princess Nourah Bint Abdulrahman University, Riyadh 11671, Saudi Arabia; 6 Department of Economics and Business, Faculty of Economic Sciences, University of Oradea, 410087 Oradea, Romania

**Keywords:** corporate social responsibility, green intrinsic motivation, tourism and hospitality, human emotions, admiration

## Abstract

Tourism and hospitality are at a crossroads. The growth and developmental potential of these industries indicate the economic benefits for an associated nation at one end. However, the environmental issues related to tourism and hospitality create challenges for the administration at another end. In most cases, a sheer amount of carbon emission in hospitality lies with energy consumption, especially electrical energy. However, past studies on environmental management have mainly focused on the supply side of energy (production) and left the terrain of the demand side (consumption by individuals) unattended. Recently, behavioral scientists have indicated that corporate social responsibility (CSR) actions of a firm can promote sustainable behavior among individuals, including employees. We tend to spark this discussion from an energy consumption perspective by investigating the relationship between CSR and energy-related pro-environmental behavior of employees (EPB) in the hospitality sector of a developing country (Pakistan). To understand the underlying mechanism of this relationship, this study proposes the mediating role of green intrinsic motivation (GIM) and the moderating role of human emotions, e.g., employee admiration (ADM). We developed a theoretical model for which the data were gathered from different hotel employees with the help of a questionnaire. We used structural equation modeling for hypotheses testing. The empirical evidence indicated that CSR significantly predicts EPB, and there is a mediating role of GIM. The study also confirmed that ADM moderates this relationship. The findings of this study will be helpful for hotel administration to understand the profound importance of CSR-based actions to promote energy-related sustainable behavior among employees, e.g., EPB. Other implications for theory and practice have been highlighted in the main text of this draft.

## 1. Introduction

Over time, climate change has emerged as a pressing issue for the global economy. The increased level of carbon emission associated with different business sectors creates a challenging situation to deal with this epic for sustainable business growth and to have a carbon-free future for this planet [1]. The escalating speed of global warming, unpredictable weathers, air and water pollution, droughts, and floods are some of the harsh realities of this modern age as an outcome of climate change [2]. The escalating pace with which this planet has been warming creates a worrisome situation for all stakeholders, including policymakers, academicians, scholars, and others. Consequently, the discussion on how to reduce carbon emissions has received escalating attention from scholars all over the world [3]. Leading from the front, the UN environmental data strongly suggests that to have a healthy and livable environment on Earth, the rising temperature has to be limited by 1.5 degree Celsius compared to the pre-industrial age [4]. As per the latest report by the Intergovernmental Panel on Climate Change (IPPC), the annual carbon emissions have reached their highest levels [5]. The same IPPC report strongly suggests that emergency measures are necessary at different levels to reduce emission level worldwide. Another report by IPPC in 2022 posits that if not managed carefully, the sever climatic conditions in different regions of the world will be a real threat to this planet in the near future [6]. In that regard, regrettably, the planet Earth has already become warmer than 1.1 degrees Celsius, indicating that urgent measures have to be taken at all levels if this planet is to have a decarbonized future in 2050 [7]. Realizing the epic nature of climate change, different steps have already been taken at a worldwide level to mitigate the effect of climate change. However, much work is still required.

The biggest culprits contributing to the current climatic situation are the energy sector, transport industry, construction, and agriculture. However, the energy sector alone is responsible for contributing more than 70% to the carbon emission levels worldwide [8]. To further complicate the situation, recent environmental data related to the energy sector indicate that in 2021, there has been an increase of 6% in global carbon emissions associated with the energy sector has been recorded. Indeed, during the last year, the energy sector’s carbon footprint reached the highest level in world history (more than 36 billion tons), offsetting the carbon decline achieved during the COVID-19 pandemic [9].

Although different models and alternatives have been proposed by the extent environmental scientists to reduce the environmental footprint of the energy sector, for example, renewable energy [10], and green energy [11,12], etc., a critical gap still exists in this debate on environmental protection via reducing the carbon print of energy sector. This issue lies with the investigation approach of previous scholars toward energy efficiency. To this end, most environmental scientists have proposed energy efficiency solutions addressing the supply side (production of energy) [13,14], whereas the demand side (consumption of energy) has been left unattended. We feel it is critical to spark the debate on environmental issues, especially related to energy consumption by individuals, because the recent environmental evidence indicates that almost 18% of energy has been used in buildings [8]. This indicates the severity of addressing this issue at an individual level. However, this is not the only reason to carry out this research study because recent literature specifies the critical importance of individuals’ energy-efficient behavior to reduce the overall carbon emission of the energy sector [15,16]. When it comes to individuals, a sheer amount of energy has been used by the individuals in buildings for heating and cooling purposes. To this end, a UN report indicates that if not managed adequately, the carbon emissions related to heating and cooling may increase by 90% in 2050, which is unbearable. The above evidence provides sufficient support to highlight the importance of promoting energy-efficient, sustainable behavior of individuals, which is identified in the literature as energy-efficient pro-environmental behavior EPB [17]. Therefore, the prime purpose of this research study is to explore the factors that may predict EPB of individuals, especially employees, in an enterprise context.

Literature on behavioral sciences proposes that in an enterprise milieu, certain behavior of employees can be explained with the help of different factors, including enterprise-level factors [18,19] and personal factors [20,21]. In that respect, the recent behavioral sciences literature acknowledged the profound importance of an enterprise’s corporate social responsibility (CSR) actions to predict different individual outcomes [22,23,24,25]. It has even been specified that CSR actions taken by an enterprise could significantly determine the pro-environmental behavior of employees [26,27]. Still, the literature on the CSR–EPB relationship is sparse. Therefore, this research intends to spark the existing body of knowledge by investigating the above-proposed relationship.

While CSR is a potential predictor at the level of an enterprise to predict employee behavior, the role of individual factors, including different psychological and emotional factors, is also important in shaping different employee outcomes. For example, the existing body of research indicates that green intrinsic motivation (GIM) of employees (a psychological factor) can significantly determine the general pro-environmental behavior of employees. Specifically, the mediating role of GIM in explaining the underlying mechanism of employee behavior was recently discussed [28,29]. In line with this literature stream, we propose that the manifestation of GIM as a mediator can be helpful in understanding the underlying mechanism of why and how CSR predicts EPB in an enterprise milieu.

From an emotional perspective, the existing body of research on employee behavior highlights that there is a definite role of human emotions in influencing behavioral outcomes [30,31]. Precisely, the literature on the emotional perspective of individuals specifies the moderating role of emotions in influencing behavior [32]. For example, a discussion exists concerning whether engagement (an emotional factor) can have a moderating effect on buffering the pro-environmental behavior of individuals [33]. Advancing this debate in a CSR context, we propose the unique role of employee admiration (ADM), which is the emotional aspect of an individual, as a moderator between the mediation path of CSR and EPB via GIM.

There are three major aims to carry out this investigation. Firstly, we aim to investigate the relationship between CSR and EPB in the hospitality sector of Pakistan. Secondly, we aim to provide the explanation how and why CSR influences EPB. To meet this aim, we propose a mediator (GIM) and a moderator (ADM) to understand the underlying mechanism of the CSR–EPB relationship. Thirdly, our aim in this investigation is to highlight the important role of employee emotions in behavior formation. Specifically, we tend to highlight how ADM (an emotional factor) helps hospitality employees in developing positive attitudinal and behavioral intentions (for example, EPB).

Our study makes the following theoretical contributions. In the first place, our study is one of the limited studies to investigate how CSR influences EPB in the hospitality industry of Pakistan. To this end, previous evidence mainly establishes a relationship between CSR and general aspects of individual pro-environmental behavior [34,35]. However, our study uniquely contributes to this discussion by proposing that CSR as an enabler to spark energy-saving ecological behavior of employees, which is worthwhile to investigate especially by considering the outsized environmental impact of the energy sector on the overall carbon emission worldwide. In the second place, our research uniquely contributes to the literature on carbon emission and environmental management by highlighting a critical gap that lies on the energy consumption side. Recently, a plethora of previous studies have proposed different energy-efficient models which focus mainly on the supply side, for example, how to produce clean and green energy [36,37]. Although these studies were important from a sustainability perspective, neglecting the consumption side of energy, especially electrical energy, was unwise. From this standpoint, this study intends to contribute to the existing body of research especially relevant to carbon emission prevention in the energy segment by highlighting the seminal role of employees. In third place, our research significantly adds to the literature on tourism and hospitality management from a sustainability perspective. In this respect, previously, most of the research related to the environment or pollution management was conducted in a manufacturing context [38,39]. Though, the manufacturing sector creates a large and more direct environmental impact on the biosphere with different industrial practices. However, considering the indirect effect of services on the biosphere, it was worthwhile to consider the services segment. To this end, our research considered the tourism and hospitality sector, which is one of the service segments creating a larger negative impact on the environment. In the fourth place, this research is unique as it helps to understand the underlying mechanism of why CSR improves the EPB of employees. To understand this mechanism, this research proposes the mediating role of GIM and moderating role of ADM in a CSR framework. Previously, such kinds of underlying mechanism did not exist to understand how and why CSR predicts EPB of employees.

## 2. Literature

The theoretical support of this study originates from the theory of social identity (SI), which is one of the important theories to explain the underlying logic of certain human behaviors, including that of employees. The social psychologist from Poland proposed this theory by arguing that human insights and identities are significantly affected by different factors, including other human beings, social and contextual factors, etc. Applying the foundational concept of this theory to the current work, we argue here that the manifestation of organizational ethics in a socially responsible enterprise can be a source to infuse positive emotions among employees, which then become the reason for a strong social identification between employees and a certain organization [40,41]. Precisely, employees enthusiastically consider their organization’s ethical perspective, especially for the welfare of all stakeholders (including internal and external). Indeed, scholars believe that the CSR actions taken by an ethical enterprise are one of the core reasons why employees show a strong identity with a socially responsible enterprise [42,43]. Specifically, Hur, et al. [44] further explained why employees are expected to show a strong level of social identity with an ethical enterprise by regarding it as “sense-making” process on employees’ part. They expressed that the CSR actions of an enterprise make sense to the employees for self-motivation and commitment to an ethical enterprise, which ultimately determine their social identity. In this respect, an ethical enterprise takes different sustainability-related initiatives to have a better, sustainable, and carbon-free environment. For example, such enterprises take different green initiatives (using clean and green energy, using eco-friendly equipment, using machines that use less electricity, etc.) to reduce their negative impact on the environment [17]. To this end, a positive CSR-PEB relation already exists in past literature [34,45]. We spark this discussion from an energy-efficient behavior perspective of employees by arguing that CSR can have a seminal role in predicting the EPB of employees in an enterprise milieu. In that regard, when employees see an ethical enterprise showing a strong concern toward energy efficiency as a part of its CSR strategy, employees are expected to reciprocate positively. Hence, by following the basic crux of SI, employees show a strong commitment to inducing the social identity of their social group (the enterprise) by adopting different behaviors towards energy efficiency. Recent scholars have also indicated that CSR can predict a target-specific aspect of PEB, e.g., EPB [16,46]. Hence, we expect:

**H1:**  *CSR can positively be linked with the EPB of employees in an enterprise*.

Motivation, especially the intrinsic level of motivation, is described as an inner motivational factor of a person that keeps him or her engaged to perform a certain task or to show a certain behavior for inner satisfaction [47]. We modify the definition of intrinsic motivation by Deci and Ryan [48] with respect to this particular study. In that regard we describe GIM by stating that it a process in which a person displays environmental concerns while he or she intends to complete a particular task in an enterprise. Further, a person with a higher level of GIM shows an increased level of commitment to preserving the environment for his/her inner satisfaction and not for external rewards. Hence, the literature on CSR and enterprise management indicates that CSR-based actions of an enterprise can inculcate positive feelings among employees [49,50]. More specifically, it is mentioned in the literature that CSR is well placed in employee psychology to boost their inner feelings, including intrinsic motivation [44,51]. Sparking the discussion on CSR and intrinsic motivation relationship, a recent study by Hao et al. [52] mentioned that an enterprise’s CSR strategies could drive employees’ intrinsic motivation, which then urges them to partake in different welfare programs for the collective welfare of others.

The sustainability initiatives of an enterprise as a part of its CSR strategy become a reason for sparking the feelings of GIM among employees, which in return motivates them to perform different sustainability-related actions to support their enterprise. One such sustainability-related action is EPB of employees to reduce energy consumption by performing different acts, for example, using less electricity in peak hours, not using heating and cooling devices (air conditioners, heaters, etc.) frequently, switching off the electric lights which are not in use, and likewise [46]. It is established in the existing body of research that intrinsic motivation, as an outcome of CSR, can mediate between CSR and different employee outcomes. For example, Loor-Zambrano et al. [53] mentioned that internal motivation could mediate between CSR and organizational commitment. Hur et al. [44] reported a mediating effect of intrinsic motivation between CSR and employee creativity. Kim, et al. [54] mentioned that intrinsic motivation significantly mediated CSR and innovation. We tend to spark this stream of literature by arguing that CSR can determine the GIM of employees, which, as a mediator, can influence a specific energy-related behavior of the employee, for example, EPB. Therefore:

**H2:**  *CSR-based actions of an enterprise can positively predict the GIM of employees*.

**H3:**  *There is a mediating role of GIM between CSR and EPB*.

The existing body of research acknowledges ADM as an emotional perspective of human behavior. We define ADM in accordance with the conception of Roseman et al. [55], who believed that ADM is an emotional judgment of a person about an entity (the enterprise, for example) in response to some specific actions of that entity. The literature on human psychology and behavior management strongly acknowledges the potential role of human emotions in predicting various individual outcomes. For example, Romani et al. [56] acknowledged the role of gratitude (a kind of emotion) in predicting the advocacy behavior of individuals. Similarly, other scholars have argued that human emotions can determine a person’s different attitudinal and behavioral intentions [57,58]. 

From a CSR standpoint, the existing literature identifies CSR as an enabler of different positive emotions among employees. For instance, Duthler and Dhanesh [59] mentioned that CSR could spark the engagement of employees significantly. Similarly, scholars like Pérez and Rodríguez del Bosque [60] mentioned that CSR not only directly predicts the loyalty level of individuals, but it also influences human emotions, which then indirectly influences the loyalty level of individuals. The conditional indirect role of personal emotions in a CSR framework was discussed previously. For example, Castro-González, et al. [61] argued that CSR induces individual emotions, which then create a buffering effect to spark individual behavior. Similarly, Xie et al. [62] highlighted the conditional indirect role of emotions in predicting extra-role behavior of individuals (EPB is also an extra-role behavior). 

Because there is a seminal role of personal emotions in influencing a certain behavior, and there is sufficient literature available that indicates that CSR can influence human emotions positively, we tend to extend this debate by arguing that CSR induces employee admiration for an ethical enterprise, which then provides buffering support to influence the mediated relationship of CSR and EPB via GIM. Therefore:

**H4:**  *CSR actions of an enterprise can predict ADM of employees in an enterprise*.

**H5:**  *There is a conditional indirect role of ADM between the mediated relationship of CSR and EPB via GIM*.

**Figure 1:**
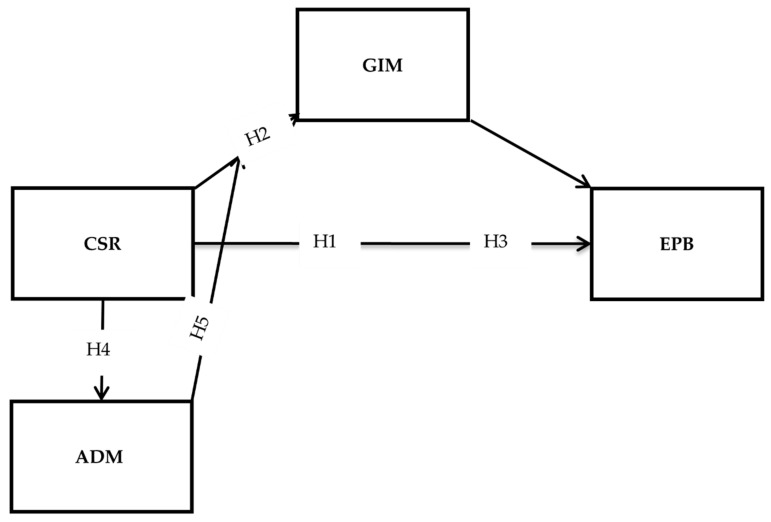
The hypothesized model.

We report the hypothesized framework of this study in Figure 1. This includes four main variables (excluding the interaction term), CSR, EPB, GIM, and ADM. CSR is the main predictor, and EPB is the criterion. GIM has been included in this model as a mediator, and ADM joins as a moderator.

## 3. Methods

### 3.1. Data Collection Process

This research study’s target segment is Pakistan’s tourism and hospitality sector. Perhaps, among various services sectors, the tourism and hospitality sector is one of the leading sectors known for its big carbon footprint. Specifically, recent climate data shows tourism and hospitality sectors significantly contribute (8%) to global carbon emissions, which is quite huge and unbearable [63]. As part of the tourism industry, the hotel sector alone contributes 1% of the total world’s emissions. In this regard, a sheer amount of carbon emission in hotels has been associated with energy consumption. Hotels throughout the globe consume a large amount of electrical energy for cooling, heating, and lighting purposes [64].

When it comes to Pakistan (a developing South Asian country), the country has emerged as one of the leading tourist destinations places during recent years [65]. Indeed, the law-and-order situation has remained a challenge for Pakistan, undermining the true potential of the tourism and hospitality industry in the past. However, data-led evidence suggests that during recent years, there has been a lot of improvement in the law and order situation [66,67,68]. According to a world leading travel and tourist agency (Condé Nast Traveller), Pakistan was at the top of the list of the world’s most preferred holiday destinations [69]. Considering the growth and developmental evidence in this sector of Pakistan, one can establish that the tourism and hospitality sector in the country contributes significantly to Pakistan’s GDP. However, there is another perspective associated with this sector, due to which we carried out this study. That is, like other regions in the world, the hospitality sector in the country is not as green as it should be. Unfortunately, Pakistan is one of the countries that have been the worst victims of climate change in recent years. Especially the devastating flood in 2022 has created a humanitarian emergency in various parts of this country. Indeed, the country needs support from every segment of the economy for a cleaner and greener atmosphere. Especially from a service perspective, the energy-related carbon emission in the hospitality sector needs to be reduced, for which the promotion of EPB at an individual level (employees, for example) is necessary.

#### Study Area

For this study, we have targeted two large cities in Pakistan for the collection of data. The two cities are Lahore and Karachi two. Lahore is renowned worldwide for its tourist traffic. Several specific reasons for selecting these two cities for the data collection existed. First, both cities are known for poor air quality index [70] and face huge energy crises. Second, many hotels operate in these two cities due to tourism and hospitality, industrial activities, and other purposes. To actuate the process of data collection, we have begun by shortlisting the hotels with CSR plans tailored to the needs of different stakeholders. It was revealed that most of the large hotel chains had different CSR-related plans to benefit the community and other stakeholders. We have contacted the administration of such hotels to cooperate in this data collection activity for the larger interest of academia and of the field. Some hotels’ administrations responded in a positive manner. Specifically, we selected a total of seven hotels (4 from Lahore and 3 from Karachi).

The data were gathered from the employees of the selected hotels. Employees from all departments were invited to this voluntary exercise. For example, employees from general administration, kitchen-related services, customer services, and others participated in this survey. This data collection activity included employees with managerial and non-managerial ranks. The data were gathered between February and April 2022. Specifically, we approached the employees of a hotel randomly. We followed the ethical standards given in the Helsinki Declaration and mentioned by previous researchers [71,72,73,74].

### 3.2. Instrument, the Unit of Analysis and Measures

To receive responses from the respondents in this study, we used a questionnaire that was self-administered in nature. Specifically, we used a five-point Likert scale for variable items’ ratings in this survey. While measuring an individual’s attitudinal and behavioral intentions is a complex process, testing various hypothesized relationships through a single measure (for example, a questionnaire) is very common in survey research. A plethora of researchers measured different attitudinal and behavioral intentions of individuals, including employees and customers using a single questionnaire [68,75,76,77]. The statements to measure a certain variable were adapted from already published and reliable sources. Prior to providing the final version of our instrument to the respondents, we invited experts from the field and academia to assess the statements of a variable. This step was important to know if there was some error or ambiguity in any statement of a question [78,79,80,81]. There were two major parts in the questionnaires related to the socio-demographic detail of respondents (for example, age, gender, experience, etc.) and variable-related questions (items). In addition, we used a multi-wave data collection (three-wave) method in this specific survey. This was purposefully done to avoid/mitigate social desirability, respondents’ fatigue, and common method variance (CMV). The unit of analysis in this study was the individual employees of a certain hotel.

To measure CSR, we used one of the very famous scales prepared by Turker [82]. This scale has a special recognition to measure CSR-related perceptions of different stakeholders, including employees and consumers. The original instrument consisted of seventeen statements. However, considering the nature of this study, we only included twelve employee-related and general CSR-related questions. The sampled items included “Our hotel encourages us to participate in voluntary activities” and “Our hotel implements different programs to mitigate the negative effect on the natural environment”.

EPB was quantified by employing the reliable scale by Blok, et al. [83], who developed a multi-dimensional scale to measure the pro-social behavior of individuals. However, as the purpose of this study was to see the CSR impact on a specific pro-environmental behavior (energy consumption related), we included eight items to measure the EPB of employees. Some items from this instrument were “While leaving my office, and there is no one else, I switch off the lights” and “I make sure that cooling/heating is off or reduced outside working hours”.

GIM was operationalized by considering the six statements by Li, et al. [29]. Indeed the original scale was developed by Amabile, et al. [84]. However, the above authors produced a modified version with respect to GIM. One item from this instrument was “I enjoy coming up with new green ideas.” Lastly, the variable ADM was quantified in this study using five statements from the work of Sweetman, et al. [85]. One item from this instrument was “I feel admiration when I think about our hotel”. The detailed list of items used in this survey is given in Appendix A.

### 3.3. Sample Size and Data Cleaning

The possible recommended sample size in this study was estimated by using the famous online calculator prepared by Dniel [86]. This application estimates a possible sample size for a specific study after taking into consideration the observed and unobserved variables, the estimated effect size, and *p*-value. One major characteristic of this application is that it estimates a study-specific sample size, especially for structural equation modeling. When provided the required input, the calculator showed that the minimum recommended study-specific sample for this study should be 233. However, to achieve a larger sample beyond 233, we distributed 450 questionnaires among hotel employees. After three phases of independent data collection intervals, we were able to receive 348 responses. However, after data screening (missing data, outliers, etc.) we deleted 29 responses and finally included 319 responses in the final dataset. For more detail on sample cleaning, Table 1 and Table 2 can be observed. Lastly, regarding the socio-demographic detail, males and females both participated in this study, however the contribution from male employees was higher (68%) than from females. Similarly, the ages of most employees were between 18 to 45 years (89%). The sampled employees had job experience, which mostly ranged from 4 to 10 years (79%). Although we included different hotel employees from different departments in this study to collect survey information, however, deciding about sample representativeness was not possible. This is because the hotel administration of most hotels was reluctant to sharing with us their employees’ stats. They were just kind enough (for certain policy-related and other reasons) to allow us to communicate directly with their employees. Therefore, commenting on sample representativeness was challenging in the absence of employee statistics (for example, how many employees were there in a particular department).

### 3.4. Common Latent Factor

Although we collected the data from employees in multiple independent intervals, we still performed a common latent factor analysis (CLF) to cross verify the manifestation of CMV. For this, we prepared two measurement models consisting of the original model and a model which a CLF contrasted. Both models were assessed to detect any significant deviation in standardized factor loadings. We realized that there were minor differences in the factor loadings of the two models. Specifically, the inclusion of a CLF into the measurement model created a variation in factor loadings. However, no factor loading showed a significant variation (beyond 0.2). This confirmed that the inclusion of a CLF into the measurement model did not create any significant variation, implying that a CMV was not a critical issue in this research.

## 4. Results

### 4.1. Preliminary Results

We performed various statistical tests in the preliminary data analysis phase. For example, we confirmed the validity and reliability of the variables in this study by examining the average variance extracted (AVE) and composite reliability test. To achieve this objective, in the first place, we examined the factor loadings of all variable items. It was revealed that no factor loading was below 0.7 which shows that all values were well-above the minimum acceptable range (≥0.5) [87,88,89,90]. This was important to see whether a sheer amount of variance in a particular variable is explained by a variable item or the error term. To explain further, the factor loading of 0.74 (first item of CSR) shows that almost 55% of variance in CSR is explained by this particular item. The formulae given in equations 1 and 2 were helpful in calculating AVEs and composite reliability.

(1) $$\mathrm{AVE}=\frac{\sum_{i=1}^{k}\lambda_{i}^{2}}{\sum_{i=1}^{k}\lambda_{i}^{2}+\sum_{i=1}^{k}.var\left(\varepsilon i\right)}$$

(2) Composite reliability=((∑λi)2/(∑λi)2+∑var(εi))

The output of the AVE analysis revealed that all AVEs were significant (>0.5). In precise, these values ranged from 0.58 (GIM) to 0.72 (ADM). This confirmed that the validity, especially convergent validity, was statistically significant. Similarly, composite reliability analysis indicated that all variables had values beyond 0.7, which showed that composite reliability values were significant [91]. This empirically verifies the inter-item consistency of a particular variable’s items. For example, the variable CSR achieved a value of 0.96, and GIM achieved a reliability score of 0.89. Table 3 can be observed for further detail.

Accordingly, we assessed the superiority of our base model (four-factor) by developing different measurement models with different compositions. In specific, we drew three alternate models (models 2, 3, 4). For example, model 4 was developed by combining all items onto a single factor. Model 2 was a two-factor alternate model and model 3 was a three-factor model. We observed different model fit indices for decision-making. For example, RMSEA, *χ*^2^/*df*, GFI, TLI, IFI, and CFI were brought into consideration to decide the goodness of model fit. The statistical evidence revealed that only the original four-factor hypothesized model was to produce superior results in all aspects (RMSEA = 0.062, and *χ*^2^/*df* = 2.88, GFI = 0.93, TLI = 0.91, IFI = 0.92, CFI = 0.91). We refer to Table 4 for more detail.

We finished the preliminary data analysis phase by assessing correlations among variables in this study. For this purpose, we observed different variable pairs and found that a significant correlation existed in each case. Correlation values were between 0.42 (EPB<=>GIM) to 0.59 (CSR<=>ADM). This shows that the variables in this study were positively related to each other, providing initial support to hypotheses statements. Moreover, no correlation result contained a critical value (0.8 or above), meaning that the multicollinearity issue was negligible in this analysis. Finally, we also assessed divergent validity (bold values in Table 5) and found that divergent validity was significant in each case. This helped us to establish that the items of one variable were dissimilar from the items of other variables.

### 4.2. Main Results

After performing several initial statistical tests, we were able to conduct the structural analysis for hypotheses testing purposes [92,93,94,95]. In that regard, we used SPSS and AMOS software to build a structural model for this study. We ensured that the preliminary assumptions to perform this structural analysis were maintained. For example, the data normality was assured, for which we observed the skewness and kurtosis values. Similarly, CSR and ADM were mean-centered in this structural analysis. Moreover, an interaction term (CSR_x_ADM) was also developed for conditional indirect analysis. Most importantly, bootstrapping sample of 5000 was used in this analysis to see the significance of indirect and interacting effects. Lastly, we observe Model 7 given in PROCESS-Macro developed by Hayes [96] to calculate conditional effect in AMOS through a user-defined estimand method.

For the convenience of readers, we report the structural analysis in three parts. First of all, we report the direct effects (H1, H2, H4). To this end, we observed that CSR was able to positively predict the EPB of employees (beta = 0.59, *p* < 0.05, CI = 0.37, 0.76). Hence, we confirm that the theoretical statement of H1 receives statistical support. Similarly, H2 and H4 also received statistical support, and we established that H2 and H4 were also significant in this analysis.

In the second stage, we report the mediation results. In this respect, the statistical evidence suggests that GIM partially and significantly mediates the CSR-EPB relationship (CSR→GIM→EPB = 0.39, *p* < 0.05, CI = 0.16, 0.44). Bootstrapping results were significant in this respect. Hence, H3 was also significant.

We report the conditional effect result of ADM in the third phase. To this end, we realized that the conditional indirect effect of ADM between CSR and GIM was significant (beta = 0.43, *p* < 0.05, with non-zero CI values). This confirmed that the existence of ADM in the structural model creates a significant buffering effect in the mediated relationship of CSR and EPB via GIM. Hence, H5 was confirmed as significant in this study. Please refer Table 6 for hypothesis results. 

## 5. Discussion

Based on the statistical outcomes, we are able to discuss these results in line with the specific objectives of this study. In that regard, the primary objective of this study was to investigate whether CSR-based actions of a hotel enterprise can determine a specific aspect (energy-related) of the pro-environmental behavior of employees. Our statistical results indicated that CSR positively predicts the EPB of employees of a certain hotel (beta = 0.59). This shows that CSR-based actions taken by an ethical hotel enterprise, especially from a sustainability perspective, can motivate employees to be engaged in EPB to meager the negative environmental impact of their hotel enterprise on the biosphere. Indeed, the manifestation of a CSR plan can be a source to infuse positive emotions among employees, which then becomes the reason for a strong social identification between employees and a specific hotel which is in line with the basic assumption of social identity theory. Specifically, the CSR actions of an ethical hotel justify the reasons why employees show a strong identity with a socially responsible hotel enterprise. Moreover, CSR actions of an enterprise make sense to the employees for self-motivation and commitment, which in return, influence their social identity. The sustainability initiatives taken by an ethical hotel, e.g., using clean and green energy, using eco-friendly equipment, and using machines that use less electricity to reduce its negative impact on the environment, are well taken by the employees. In response to such sustainability initiatives as a part of CSR, employees also become self-responsible, and following the crux of social identity theory, they support the sustainability perspective of their hotel by taking different energy-saving initiatives on their part. Therefore, our results confirm that CSR significantly determines the EPB of employees. This finding is in line with previous behavioral scientists [16,46].

Another important purpose of this study was to understand the underlying mechanism of the CSR-EPB relationship with the help of GIM as a mediator (beta = 0.39). Although the existing body of research indicates that CSR influences different individual outcomes, it is important to explain the underlying mechanism of the above-proposed relationship. To this end, our results confirmed that CSR, at one end, creates a positive effect on the GIM of employees (beta = 0.46). At another end, GIM, as an antecedent of CSR, mediates between CSR and EPB to explain the underlying mechanism of the above association. Specifically, when employees observe different CSR-related green initiatives of their ethical hotel for the welfare of all stakeholders, it positively induces their intrinsic motivation, especially GIM. The available discussion on CSR and employee behavior management already indicates that CSR-based actions of an enterprise can inculcate positive inner feelings among employees, including inner motivation [44,51]. In line with this research stream, our research indicates that CSR can determine the GIM of employees, which then mediates between CSR and EPB. This is in line with previous studies in which different behavioral scientists acknowledged the mediating role of intrinsic motivation in explaining the logic of certain employee behavior in an enterprise context [44,53].

Lastly, our research highlights the important role of human emotions, especially ADM, in buffering the mediating relation of CSR and EPB via GIM (beta = 0.43). Past literature acknowledges the profound importance of human emotions in deriving different attitudes and behavior [30,31]. Extending this discussion from an ADM standpoint, our research proposes that the CSR actions of an ethical hotel are well-observed by employees. Specifically, employees feel admiration to associate themselves with an ethical enterprise which shows a collective caring concern for all stakeholders. This argument also receives empirical support from previous scholars who proposed that CSR can positively impact human emotions [59,60]. All in all, our results confirm that CSR positively influences the ADM of employees, which then creates a buffering effect between the mediated relationship of CSR and EPB through GIM. The conditional indirect effect of human emotions in a CSR framework was also highlighted by Xie, et al. [62].

### 5.1. Implications for Theory

Altogether, our study makes the following theoretical contributions to the already existing literature on CSR and employee behavior. Firstly, our study is one of the limited investigations to investigate how the CSR-based actions of a hotel can be helpful in influencing a specific aspect of pro-environmental behavior, for example, the energy-consumption behavior of employees. In this regard, previously different behavioral scientists were able to provide empirical evidence that CSR predicts the general aspect of employees’ pro-environmental behavior [34,35]. However, our study uniquely contributes to this discussion by proposing that an organization’s CSR actions can spark employee energy-saving behavior, which is very important to investigate considering the outsized environmental impact of the energy sector on overall carbon emission worldwide.

Secondly, our research uniquely contributes to the literature on carbon emission and environmental management by highlighting a critical gap that lies on the energy consumption side. In this regard, many previous studies have proposed different energy-efficient models which focus on the supply side of clean and green energy [36,37]. Although these studies were important from a sustainability perspective, neglecting the consumption side of energy, especially electrical energy, was unwise. From this standpoint, this study intends to spark the existing body of research especially relevant to carbon emission prevention in the energy segment by highlighting the seminal role of employees.

Thirdly, our research significantly adds to the literature on tourism and hospitality management from a sustainability perspective. In this respect, previously, most of the research related to the environment or pollution management was conducted in a manufacturing context [38,39]. Though the manufacturing sector creates a larger and more direct environmental impact on the biosphere with different industrial practices, considering the indirect effect of services on the biosphere, it was worthwhile to consider the services segment. To this end, our research considered the tourism and hospitality sector, which is one of the service segments creating a larger negative impact on the environment.

Lastly, this research is unique as it helps to understand the underlying mechanism of why CSR improves the EPB of employees. To understand this mechanism, this research proposes the mediating role of GIM and moderating role of ADM in a CSR framework. Previously, such a kind of underlying mechanism did not exist to understand how and why CSR predicts the EPB of employees.

### 5.2. Implications for the Hospitality Sector

Our research is also important for practice, especially in the administration of the hospitality sector. In this regard, our research helps the administration of a certain hotel to realize the profound importance of CSR-based actions, especially sustainability-related actions to determine energy-related sustainable behavior of employees, e.g., EPB. This implication is special to the hospitality sector, especially in Pakistan. Considering the larger environmental impact of this sector, it is worthwhile for the administration to take every measure to protect the environment and biosphere. Moreover, because most of the electrical energy in hotels is consumed for heating, lighting, and cooling purposes, it is really important for employees to understand their seminal in reducing the overall energy consumption of a hotel organization for which there is a definite role of CSR-based actions.

The management of a hotel needs to carefully plan CSR strategies, especially from a sustainability perspective, because such steps not only improve the supply side of energy (e.g., using energy-efficient equipment or installing solar energy production panels), it also improves the consumption side of energy by promoting the energy efficient pro-environmental behavior among employees. Similarly, the hotel administration needs to understand that CSR not only directly predicts EPB of employees, but also explains this phenomenon by influencing different psychological factors (e.g., GIM) and human emotions (e.g., ADM), which then explain the underlying logic why employees are involved in a certain sustainable behavior to preserve the eco-systems for a better and cleaner future.

## 6. Conclusions

Tourism and hospitality are at a crossroads. The growth and development potential of these industries indicate the economic benefits for a nation associated with the tourism and hospitality sector. However, the environmental issues related to tourism and hospitality are challenging for hotel administrations. Considering the increasing level of awareness regarding climate change among individuals and by considering the role of different segments of an economy in preserving the biosphere, it is important for the administrations of certain hotels to mitigate their environmental footprint by opting for different environmental strategies at every level. To achieve such sustainability objectives, there is a profound role of CSR-based actions taken by an ethical hotel organization. Especially from an energy consumption perspective, it is important for hotel administrations to promote the EPB among employees, for which CSR represents a way forward, as suggested by the empirical results of this study.

To conclude, we suggest that hotel administrations carefully plan and execute CSR policies in light of the sustainability context. To this end, hotel administrations are suggested to closely align their CSR plan with a sustainability perspective for a better and carbon-free future. Moreover, we suggest that hotel administrations carefully align different employee training and developmental plans with their CSR strategy. As an example, we hope our study will help various hotel administrations better understand the importance of human factors. A further example would be for hotel administrations to act upon the potential that motivation and emotions have upon employees and to promote EPB. Positive emotions elicited among employees in response to CSR may motivate hospitality employees to deliver their best to an ethical hotel organization. This eventually enables a hospitality organization to better serve the customers, improving the ultimate performance of a hotel.

In this regard, hotel administrations need to merge employee training programs and place them under the umbrella of CSR to promote their inner motivation and commitment level to partake in different sustainability initiatives, especially related energy preservation and consumption.

Moreover, this research faces a few limitations, which illuminate the way of future behavioral sciences scholars to conduct research in the same area. At first, the data for this study were gathered from Karachi and Lahore. Although considering the poor environmental conditions in these cities and considering the tourism and hospitality potential of these cities, it was important to collect data from these cities. However, we propose to include more geographical areas in the future so that the drawn results may reflect better generalizability. Second, this study only considered employees for data collection. Considering that employees are important stakeholders and spend a significant amount of time in a hotel daily, it was important to carry out this research from an employee perspective. Nonetheless, we propose that in the future, it will be interesting to see how a hotel’s CSR-based actions can determine customers’ EPB. Third, this study did not apply a probability sampling method due to the unavailability of any sampling frame (the administration of hotels did not share it). Because probability sampling is more suitable for the reliability of causal relationships, we suggest in the future, if possible, a probability sampling method should be applied. Last, an interesting aspect would be to compare the answers given by various groups of interviewed persons. However, we did not produce any such comparison. Therefore, we suggest future researchers consider this point in upcoming investigations.

## Figures and Tables

**Table 1:** Data cleaning, outliers, and response rate.

Distributed	Returned	Unreturned	Removed	Outliers	Final
450	348	102	29	13	319
-	77.3%	22.7%	8.33%	4.4%	70.9%

**Table 2:** Responses identified as outliers.

Response	Mahalanobis d-Squared	p1	p2
72	18.879	0.000	0.025
286	18.879	0.000	0.000
171	11.959	0.003	0.009
39	10.648	0.005	0.021
150	10.648	0.005	0.005
402	10.648	0.005	0.001
244	10.648	0.005	0.000
60	7.740	0.021	0.228
148	7.085	0.029	0.447
88	6.556	0.038	0.664
120	6.556	0.038	0.548
197	6.116	0.047	0.645
376	6.116	0.047	0.540

**Table 3:** Summary of initial analyses.

	λ	λ^2^	E-Variance
	0.74	0.55	0.45
CSR	0.77	0.59	0.41
	0.81	0.66	0.34
AVE = 0.65	0.86	0.74	0.26
CR = 0.96	0.72	0.52	0.48
∑λ^2^ = 7.75	0.79	0.62	0.38
Items = 12	0.74	0.55	0.45
	0.80	0.64	0.36
	0.88	0.77	0.23
	0.77	0.59	0.41
	0.82	0.67	0.33
	0.92	0.85	0.15
	0.70	0.49	0.51
EPB	0.86	0.74	0.26
AVE = 0.62	0.81	0.66	0.34
CR = 0.93	0.71	0.50	0.50
∑λ^2^ = 4.92	0.74	0.55	0.45
Items = 8	0.78	0.61	0.392
	0.83	0.69	0.31
	0.83	0.69	0.31
	0.72	0.52	0.48
GIM	0.75	0.56	0.44
AVE = 0.58	0.81	0.66	0.34
CR = 0.89	0.80	0.64	0.36
∑ λ^2^ = 3.48	0.78	0.61	0.39
Items = 6	0.70	0.49	0.51
ADM	0.89	0.79	0.21
AVE = 0.72	0.72	0.52	0.48
CR = 0.93	0.78	0.61	0.39
∑λ^2^ = 3.59	0.93	0.86	0.14
Items = 5	0.90	0.81	0.19

Notes: λ = Item loadings, CR = composite reliability, ∑λ^2^ = sum of the square of item loadings, E-Variance = error variance.

**Table 4:** Model fitness.

Model	*χ*^2^/*df* (<3)	Δ*χ*^2^*/df -*	RMSEA (<0.08)	GFI (>0.9)	TLI (>0.9)	IFI (>0.9)	CFI (>0.9)
1	2.88	_	0.062	0.93	0.91	0.92	0.91
2	5.64	2.43	0.072	0.84	0.84	0.82	0.86
3	6.92	1.44	0.101	0.67	0.62	0.60	0.64
4	8.42	1.59	0.182	0.48	0.41	0.33	0.46

Note: Model 1 = four-factor model, model 2 = three factor by combining CSR and GIM into one factor, model 3 = two factor model by combining CSR + GIM and EPB + ADM, model 4 = one factor by combining CSR + GIM + ADM + EPB.

**Table 5:** Correlations and discriminant validity.

Variable	1	2	3	4
1. CSR	0.80	0.55	0.49	0.59
2. EPB	(2.92, 0.50)	0.78	0.42	0.50
3. GIM		(3.39, 0.62)	0.77	0.44
4. ADM			(2.86, 0.49)	0.85
				(3.08, 0.57)

Notes: values in parenthesis = Mean and standard deviation, bold values = discriminant validity, *p <* 0.001.

**Table 6:** Hypotheses results.

Hypotheses	Estimates (SE)	*t/z*	*p*-Value	CI
(CSR→EPB)	0.59 (0.060)	9.83	****	0.37, 0.76
(CSR→GIM)	0.46 (0.053)	8.68	****	0.32, 0.58
(CSR→ADM)	0.55 (0.048)	11.46	****	0.14, 0.77
Indirect effect (CSR→GIM→EPB)	0.39 (0.027)	14.44	****	0.16, 0.44
The conditional indirect effect of AM on CSR→GIM→EPB	0.43 (0.033)	13.03	****	0.22, 0.49

Notes: CI = 95% confidence interval with lower and upper limits. ****, level of confidence at 99 percent.

## Data Availability

Data will be provided on demand.

## Appendix A. Variable Items Used in This Study

**CSR**
Our hotel participates in activities which aim to protect and improve the quality of the natural environment
Our hotel makes investments to create a better life for the future generation
Our hotel implements special programs to minimize its negative impact on the natural environment
Our hotel targets sustainable growth, which considers the future generations
Our hotel supports non-governmental organizations working in the problematic areas
Our hotel contributes to the campaigns and projects that promote the well-being of the society
Our hotel encourages its employees to participate in the voluntary activities
Our hotel policies encourage the employees to develop their skills and careers
The management of our hotel primarily concerns with employees’ needs and wants
Our hotel implements flexible policies to provide good work and life balance for its employees
Our hotel decisions related to the employees are usually fair
Our hotel supports employees who want to acquire additional education
**EPB**
I check whether thermostats are set correctly in my office
I wear more/less clothes instead of putting the heating/air conditioning on.
I make sure that heating/airconditioning is off or reduced outside working hours
I reduce heating/air conditioning in unused rooms.
I switch off electricity devices (computer/notebook, etc.),when I leave my office for a considerable period
I switch off electricity devices (computer/notebook, etc.) when I go home
I switch on the lights when I come to the office in the morning and switch them off while leaving
When I leave my office for a considerable period of time, and there is no one else, I switch off electricity devices
**EMEC**
I really care about the environmental concern of our hotel
I would feel guilty about not supporting the environmental efforts of our hotel
The environmental concern of our hotel means a lot to me
I feel a sense of duty to support the environmental efforts of our hotel
I really feel as if our hotel’s environmental problems are my own
I feel personally attached to the environmental concern of our hotel
I feel an obligation to support the environmental efforts of our hotel
I strongly value the environmental efforts of our hotel
**GIM**
I enjoy coming up with new green ideas
I enjoy trying to solve environmental tasks on the job
I enjoy tackling with environmental tasks that are completely new
I enjoy improving existing green ideas on the job
I feel excited when I have new green ideas
I feel like becoming further engaged in the development of green ideas
**ADM**
I feel admiration when I think about our hotel
I feel respect when I think about our hotel
I feel inspired when I think about our hotel
CSR activities of our hotel amaze me
CSR activities of our hotel inspire me
